# Surgical Retrieval of Grains in a Case of Cystic Actinomycetoma

**DOI:** 10.4269/ajtmh.25-0581

**Published:** 2026-03-03

**Authors:** Deepti Singh, Sowmya Susan, Sheetanshu Kumar

**Affiliations:** Department of Dermatology and STD, Jawaharlal Institute of Postgraduate Medical Education and Research, Puducherry, India

A 50-year-old female agricultural worker from South India engaged in groundnut cultivation involving prolonged barefoot work in wet fields complained of progressive, multinodular swelling over the left foot for 3 years with intermittent purulent discharge and pale granules. Examination of left foot revealed diffuse tumefaction and multiple cystic swellings and pus discharging sinus tracts (Figure 1A).

**Figure 1.**
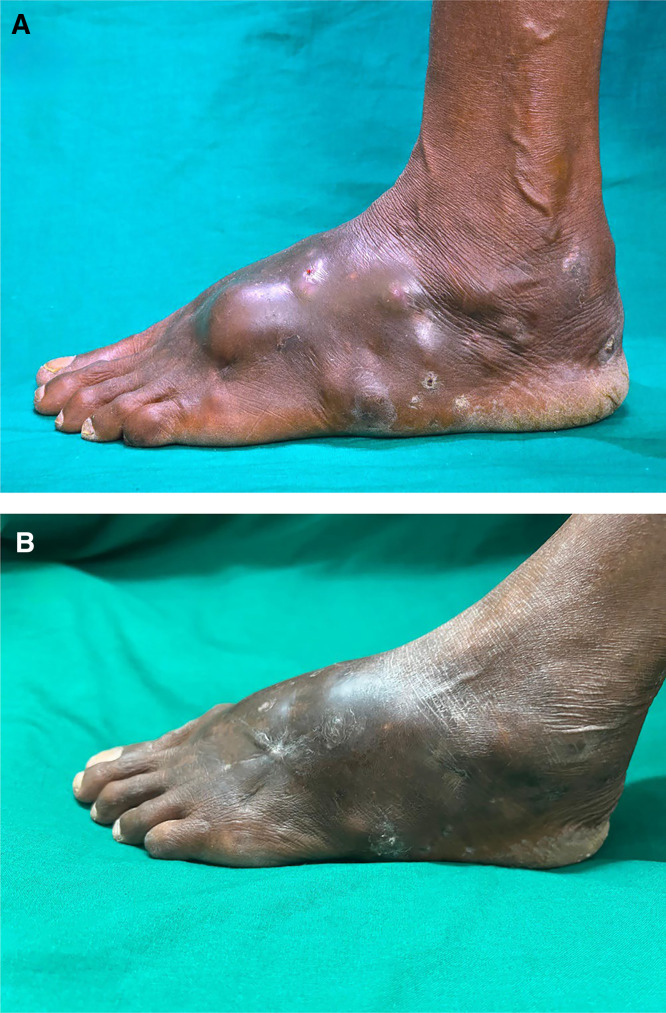
(**A**) Diffuse tumefaction with multiple cystic swellings of sizes from 1 × 1 cm to 2.5 × 2.5 cm along with a few sinus openings over her left foot at baseline. (**B**) Follow-up photograph after completion of three cycles of therapy showing marked reduction in swelling, resolution of cystic nodules, and healed sinuses.

X-ray of foot showed punched-out lytic lesions and degenerative changes involving the second and third metatarsal bones of left foot; ultrasound revealed hypoechoic collections extending into metatarsal spaces. Despite overnight saline dressings, grains could not be obtained.

During incisional biopsy, surgical exploration of one of the nodulocystic lesions yielded a cystic cavity lined by thick fibrous tissue containing multiple pale granules (Figure 2).

**Figure 2.**
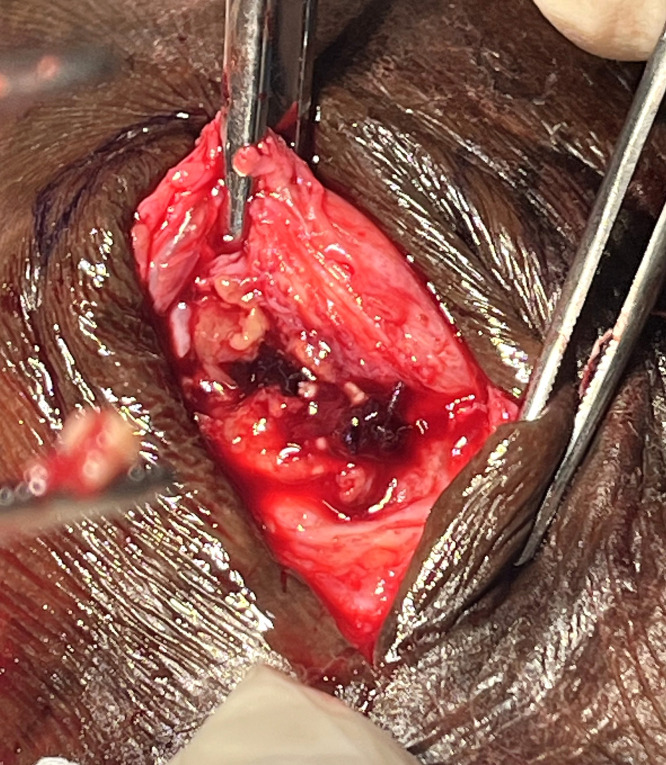
Intraoperative image demonstrating multiple pale granules retrieved from a surgically explored nodulocystic lesion and revealing a cystic cavity lined by thick fibrous tissue containing pale grains.

Microscopy of the granules revealed gram-positive, weakly acid-fast positive filamentous bacteria consistent with *Nocardia* (Figure 3). Cultures of granules performed on blood agar, Sabouraud dextrose agar, and Lowenstein–Jensen media were negative. Fine-needle aspiration cytology (FNAC) was inconclusive. Histopathology of skin biopsy showed granulation tissue with Splendore–Hoeppli phenomenon suggestive of actinomycetoma.

**Figure 3.**
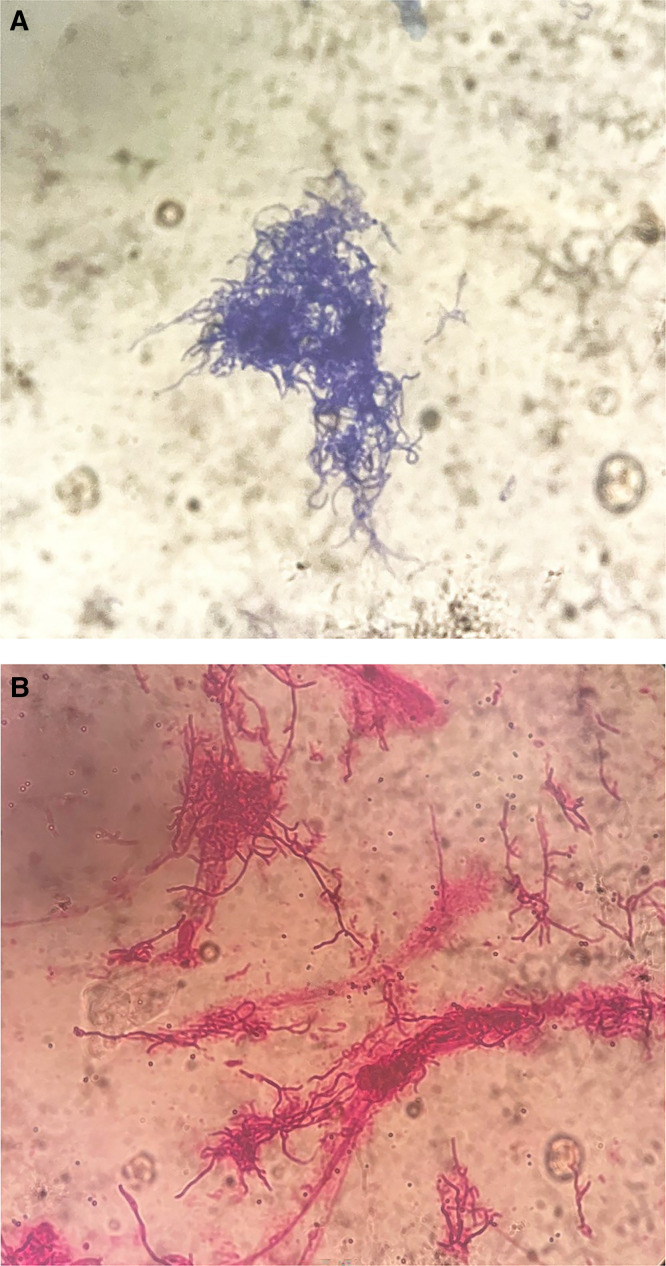
(**A**) Microscopy of crushed grains showing gram-positive filamentous bacteria on gram staining at ×100. (**B**) Microscopy of crushed grains showing weakly acid-fast positive filamentous bacteria on Ziehl–Neelsen staining at ×100.

The patient was treated with the Welsh regimen: injection amikacin 15 mg/kg intravenously divided into two doses plus tablet trimethoprim-sulfamethoxazole (7 and 35 mg/kg per day) for 3 weeks followed by 2 weeks of only tablet trimethoprim-sulfamethoxazole (7 and 35 mg/kg per day) in each cycle. This was repeated for three cycles (3 × 5 = 15 weeks). There was marked improvement at 15 weeks (Figure 1B), with reduction in swelling, resolution of cystic nodules, and healing of sinuses.

Mycetoma is a chronic granulomatous infection characterized by painless swelling, multiple sinus tracts, and discharge containing grains,^1^ and it is classified into eumycetoma (fungal) and actinomycetoma (bacterial), which require distinct treatment approaches.

Grains represent compact colonies of micro-organisms and usually help in the diagnosis of mycetoma based on clinical and microbiological characteristics. Grains are usually retrieved using techniques like overnight saline dressings, manual expression, FNAC, or ultrasound-guided aspiration. However, grain collection can be challenging, especially in deep-seated or encapsulated cystic lesions where there is no spontaneous extrusion of grains.^2^

In such cases, surgical exploration allows for direct visualization and access to cystic cavities, and it can be performed to extract grains. Surgical exploration can be performed at the site marked by prominent activity characterized by active pus discharge, history of recent grain extrusion, cystic consistency, or inflammation.

This case highlights surgical retrieval of grains as a vital diagnostic strategy in mycetoma.

## References

[1] Relhan V Mahajan K Agarwal P Garg VK, 2017. Mycetoma: An update. Indian J Dermatol 62: 332–340. 28794542 10.4103/ijd.IJD_476_16 PMC5527712

[2] Bellalah A Abdeljelil NB Njima M Hammouda SB Khalifa SB Koubaa M Zakhama A Hadhri R, 2021. Cystic form of *Actinomycotic mycetoma*: A new case with a diagnostic challenge. Clin Case Rep 9: e04064. 33936735 10.1002/ccr3.4064 PMC8077338

